# Ultrasound therapy with optimal intensity facilitates peripheral nerve regeneration in rats through suppression of pro-inflammatory and nerve growth inhibitor gene expression

**DOI:** 10.1371/journal.pone.0234691

**Published:** 2020-06-17

**Authors:** Akira Ito, Tianshu Wang, Ryo Nakahara, Hideki Kawai, Kohei Nishitani, Tomoki Aoyama, Hiroshi Kuroki

**Affiliations:** 1 Department of Motor Function Analysis, Human Health Sciences, Graduate School of Medicine, Kyoto University, Kyoto, Japan; 2 Department of Development and Rehabilitation of Motor Function, Human Health Sciences, Graduate School of Medicine, Kyoto University, Kyoto, Japan; 3 Department of Orthopaedic Surgery, Graduate School of Medicine, Kyoto University, Kyoto, Japan; Medical University Innsbruck, AUSTRIA

## Abstract

**Background:**

Therapeutic ultrasound (US) is a promising physical therapy modality for peripheral nerve regeneration. However, it is necessary to identify the most effective US parameters and clarify the underlying mechanisms before its clinical application. The intensity of US is one of the most important parameters. However, the optimum intensity for the promotion of peripheral nerve regeneration has yet to be determined.

**Objectives:**

To identify the optimum intensity of US necessary for the promotion of peripheral nerve regeneration after crush injuries in rats and to clarify the underlying mechanisms of US by mRNA expression analysis.

**Methods:**

We inflicted sciatic nerve crush injuries on adult Lewis rats and performed ultrasound irradiation using 4 different US intensities: 0 (sham stimulation), 30, 140, and 250 mW/cm^2^ with frequency (5 days/week) and duration (5 min/day). We evaluated peripheral nerve regeneration by quantitative real-time PCR one week after injury. Histomorphometric analyses and motor function analysis were evaluated 3 weeks after injury.

**Results:**

US stimulation enhanced re-myelination as well as sprouting of axons, especially at an intensity of 140 mW/cm^2^. mRNA expression revealed that US suppressed the expression of the inflammatory cytokines TNF and IL-6 and the axonal growth inhibitors SEMA3A and GSK3β.

**Conclusions:**

An intensity of 140 mW/cm^2^ was optimal to support regeneration of the sciatic nerve after a crush injury in rats by, in part, the suppression of pro-inflammatory and nerve growth inhibitor gene expression.

## Introduction

Peripheral nerves connect the central nervous system to the limbs and organs. Although peripheral nerves have intrinsic regenerative abilities, the repair process is slow. In many cases, peripheral nerve injury results in reduced quality of life because of motor disturbances and sensory deficits [1]. Interventions for peripheral nerve regeneration have been developed using tissue-engineered scaffolds and cell-based therapies [2,3]. However, each therapy has advantages and disadvantages, and the nerve-regeneration capabilities of these remedies is inferior to that of autologous nerve grafting. Therefore, researchers are currently developing other approaches for peripheral nerve regeneration. It has been reported that adequate mechanical stress promotes peripheral nerve regeneration [4]. Additionally, mechanical stress may have synergistic positive effects with regenerative therapies [5–7]. Mechanical stress may be used to stimulate peripheral nerve regeneration during neurorehabilitation.

Therapeutic ultrasound (US) is a promising treatment that promotes nerve regeneration [8–12]. In order to utilize US for peripheral nerve regeneration in a clinical setting, it is necessary to optimize US parameters and elucidate the underlying mechanisms of its effects. US intensity is one of the most important parameters, but few reports have attempted to identify the effective US intensity required to promote peripheral nerve regeneration [13–15]. Therefore, the optimal US intensity necessary to promote peripheral nerve regeneration has yet to be determined.

Low-intensity pulsed US promotes the healing of bone fractures [16] and various soft tissues including peripheral nerves [17]. Most previous reports investigating mechanisms associated with the effects of US on peripheral nerve regeneration have focused on the expression of neurotrophic factors, such as nerve growth factor (NGF) and brain derived neurotrophic factor (BDNF) [10,12]. However, to the best of our knowledge, there is no evidence of any effect of US on neurological pro-inflammatory and axonal inhibitory factors, although the anti-inflammatory effects in other tissues are well known [18]. Uncontrolled inflammation and an increase in axonal inhibitory factors such as semaphorin 3A (SEMA3A) impede nerve regeneration in central nervous system diseases [19]. The identification of the effects of US on these inhibitory factors in peripheral nerve injury will serve as novel fundamental knowledge for clinical use.

To confirm the optimum intensity and mechanisms of the effect of US, we used a rat model of sciatic nerve crush injury, which is a simple and conventional model frequently used in peripheral nerve regeneration studies. The purpose of this study was (i) to identify the optimum intensity of US required to promote peripheral nerve regeneration after a crush injury in rats and (ii) to investigate the mechanisms of US by gene expression analysis.

## Materials and methods

### Surgical induction of sciatic nerve crush injury and experimental groups

**Fig 1:**
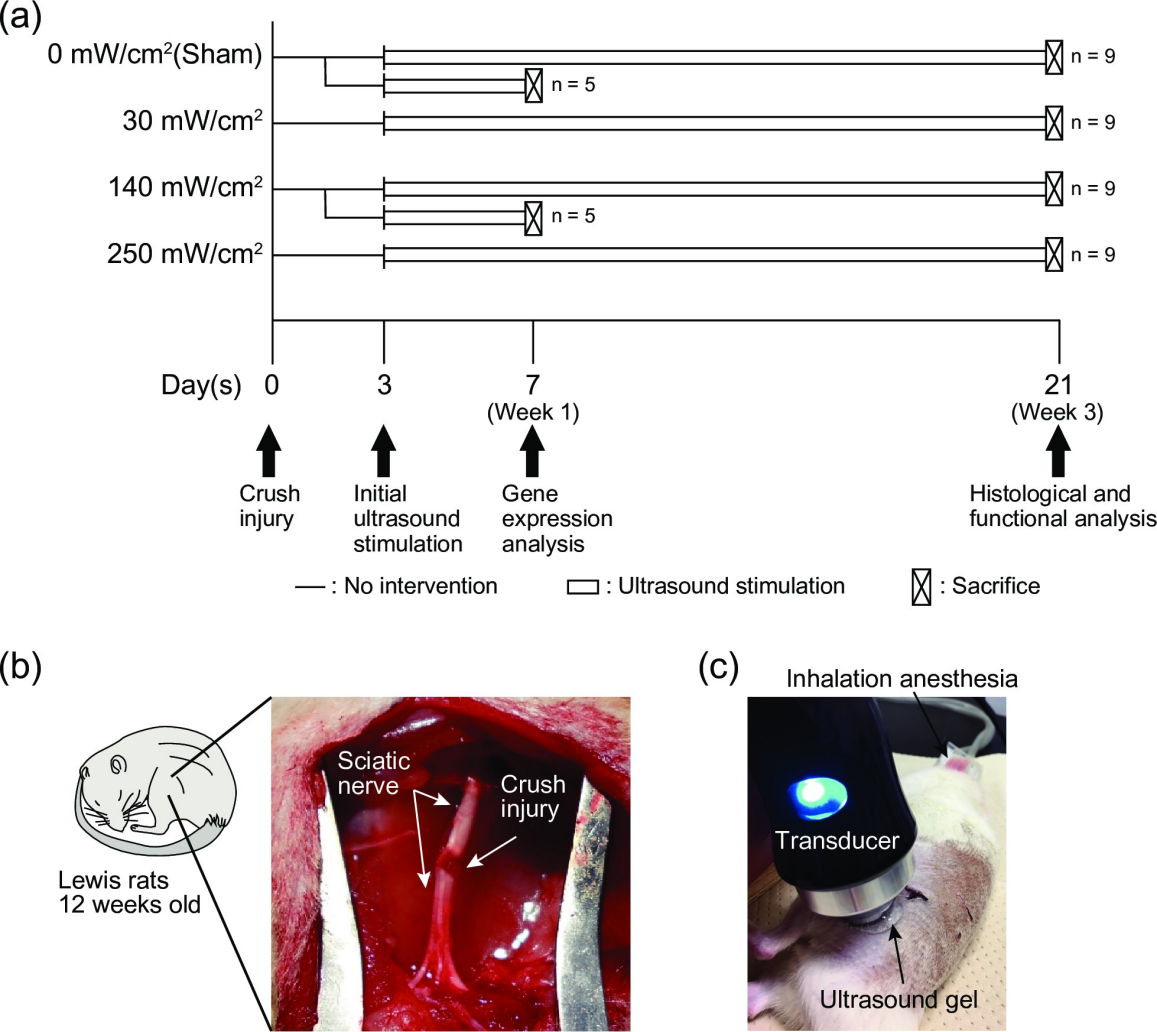
Experimental design, images of crush injury and ultrasound therapy. (a) Experimental design describing the ultrasound protocol and the analytical methods. Rats were randomly assigned to 4 groups based on the ultrasound intensity. The sciatic nerve crush injury was induced on day 0, and ultrasound stimulation was initiated on day 3. Histological and functional analyses were performed on day 21 (n = 9 per group). To clarify the mechanisms of the effect of the ultrasound, the gene expression analysis was performed for 0 mW/cm^2^ group and 140 mW/cm^2^ group on day 7 (n = 5 per group). (b) A representative image of the sciatic nerve crush injury. A 2-mm long crush injury was created in the sciatic nerve. (c) A representative image of the ultrasound therapy. Ultrasound irradiation was performed with an unfocused circular probe with a 0.9 cm^2^ effective radiation area over ultrasound transmission gel.

A total of 46 adult male Lewis rats (12 weeks old; mean body weight = 298.4 g ± 8.69 standard deviation [SD]) were purchased and placed in standardized cages (3 animals per cage) in a 12-hours light/dark cycle at 25°C on sawdust bedding. The rats were allowed to move freely in their cages and were given free access to food and water. Also, the rats were monitored for any health or welfare problems and there were no adverse events in any experimental group. Thirty-six rats were randomly divided into four groups based on US intensity (spatial average temporal average: SATA): 0 (sham stimulation), 30, 140, and 250 mW/cm^2^ (n = 9 in each group; Fig 1A). A sciatic nerve crush injury (axonotmesis) was created in the left leg using a previously reported protocol [20] under anesthesia induced with 64.8 mg/kg pentobarbital sodium (Somnopentyl; Kyoritsu Seiyaku Corp., Tokyo, Japan). Briefly, the sciatic nerve of the rats was exposed by a lateral longitudinal incision from the proximal to the mid-thigh of the left hind limb, and a 2-mm section of the nerve at the site below the gluteal tuberosity was crushed for 10 s with a needle holder (No. 12501–13, Fine Science Tools, Inc., North Vancouver, Canada) to completely rupture the nerve fibers (Fig 1B). The proximal end of the crush injury was marked at the epineurium with a 9–0 nylon suture and the surgical incision was closed with 4–0 nylon sutures. The remaining 10 rats were used for gene expression analysis. The rats underwent the same surgical procedure described above and were randomly divided into two groups; 0 (sham stimulation) and 140 mW/cm^2^ (n = 5 in each group). All procedures were approved by the Institutional Animal Care and Use Committee of Kyoto University (approval number: Med Kyo 17029).

### Ultrasound protocol

US stimulation was started 3 days after the injury according to a previous study [11]. All rats were anesthetized with 2% isoflurane and placed on their right side. An US transducer was placed on the skin at the injury site after application of an US transmission gel (Fig 1C). Ultrasonic irradiation was then performed at each intensity or turned the power off for the sham stimulation five days per week using a portable ultrasonic treatment apparatus with an unfocused circular probe with an effective radiation area of 0.9 cm^2^, and 2.9 beam non-uniformity ratio (UST-770, ITO Physiotherapy & Rehabilitation, Tokyo, Japan). The radiation parameters were as follows: 1 MHz frequency, 1 kHz repeating frequency, 20% duty cycle, and 5 min/day treatment duration. At 1 or 3 weeks after the injury, the rats were sacrificed by an over-dose of pentobarbital sodium intraperitoneal injection and exsanguination. Thereafter, the sciatic nerve was isolated for evaluation.

### Histomorphometric analyses

Three weeks after the injury, a 5-mm-long sciatic nerve specimen was dissected from the epineurium marker, and prefixed with 1.44% paraformaldehyde and 1% glutaraldehyde in 0.036 M phosphate buffer (pH = 6.8) at 4°C overnight, then fixed with 1% osmium tetroxide in 0.1 M phosphate buffer for 120 min. The specimens were then dehydrated with graded ethanol and embedded in EPON (Luveak, Nacalai Teque, Kyoto, Japan). Subsequently, 1-μm-thick transverse sections were stained with toluidine blue solution and images of the cross-sectioned sciatic nerve were obtained using a light microscope (ECLIPSE 80i, Nikon, Tokyo, Japan). Myelinated fiber density was calculated by counting the number of myelinated nerve fibers in three different random areas of 90,000 μm^2^, one cross-section per rat, accounting for at least 30% of the total area of the image using ImageJ (National Institutes of Health, Bethesda, MD) according to the previous study [20]. The results were expressed as myelinated fiber density (fibers/mm^2^).

Ultrathin transverse sections of the same tissue stained with uranyl acetate and lead citrate were also obtained using a transmission electron microscope (TEM) (Model H-7000, Hitachi High-Technologies, Tokyo, Japan). Ten pictures from one cross-section were randomly obtained at 2000x magnification, and the areas of myelinated nerve fiber, axon, and surrounding myelin were measured for at least 39 fibers per rat by tracing their margin using ImageJ. These histomorphometric analyses were performed blindly by a trained specialist.

### Sciatic functional index

Motor function recovery was assessed by calculating the sciatic functional index (SFI) three weeks after the injury. The rats’ footprints were obtained by painting each foot and allowing the rats to walk through a wooden walking alley (9 × 10 × 60 cm) covered with a sheet of paper. Three footprints were selected from clearly inked randomly chosen footprints, and three different parameters were measured: (1) distance from the heel to the third toe (Print Length; PL); (2) distance from the first toe to the fifth toe (Toe Spread; TS); (3) distance from the second toe to the fourth toe (Intermediate Toe Spread; ITS). The SFI was calculated according to the formula: SFI = -38.3 ((𝐸𝑃𝐿 - 𝑁𝑃𝐿) / 𝑁𝑃𝐿) + 109.5 ((𝐸𝑇𝑆 - 𝑁𝑇𝑆) / 𝑁𝑇𝑆) + 13.3 ((𝐸𝐼𝑇𝑆 - 𝑁𝐼𝑇𝑆) / 𝑁𝐼𝑇𝑆) - 8.8, where E is the injured side (left side) and N is the normal side (right side) as reported in a previous study [21]. A value of 0 indicates normal function and a value of -100 indicates total impairment.

### Quantitative real-time PCR

One week after the injury, a 10-mm long sciatic nerve specimen was excised from the distal portion of the injured site, and the normal sciatic nerve specimen at the corresponding location of the contralateral side was also excised as an intact control. Total RNA was extracted using the RNeasy Plus Universal Mini Kit according to the manufacturer’s protocol (Qiagen, Valencia, CA) and tested for the purity and quality using the NanoDrop2000 (Thermo Fisher Scientific, Wilmington, DE) and the 2100 Bioanalyzer System (Agilent, Santa Clara, CA), respectively. The A260/A280 ratio and the RNA Integrity Number for all samples were > 2.0 and > 8.4 (average = 9.2), respectively. Total RNA (1 μg) was reverse-transcribed to synthesize cDNA and quantitative real-time PCR was performed via the Applied Biosystems7500 Real-Time PCR System (Applied Biosystems, Foster City, CA) using the commercially available TaqMan gene expression assays (Applied Biosystems) for pro-inflammatory cytokines, axonal growth inhibitors, and neurotrophins (Table 1). Standard enzyme and cycling conditions for the 7500 Real-Time PCR System were used.

**Table 1:** TaqMan gene expression assays for real-time PCR.

Symbol	Gene name	Assay ID
*TNF*	tumor necrosis factor	Rn99999017_m1
*IL-6*	interleukin 6	Rn01410330_m1
*SEMA3A*	semaphorin 3A	Rn00436469_m1
*GSK3B*	glycogen synthase kinase 3 beta	Rn01444108_m1
*NGF*	nerve growth factor (beta polypeptide)	Rn01533872_m1
*NT3*	neurotrophin 3	Rn00579280_m1
*PSMC4*	Proteasome 26S subunit, ATPase, 4	Rn00821605_g1

To confirm a stable endogenous reference gene in this study, 32 candidate genes were evaluated using TaqMan® Array Rat Endogenous Control plate (Applied Biosystems). Reference gene ranking was performed using the NormFinder [22] and RefFinder [23]. Proteasome 26S subunit, ATPase, and 4 (PSMC4) were found to be the most stable genes in our study conditions (S1 Fig). Therefore, PSMC4 was used as the endogenous reference gene. The data obtained by real-time PCR was analyzed using the comparative threshold cycle method. Briefly, the amount of the target gene was normalized to that of PSMC4, the value of the calibration sample (intact nerve specimen) was set to 1, and the values for sham and 140 mW/cm^2^ group were shown relative to that of the calibration sample.

### Statistical analysis

All statistical analyses were performed using JMP 11 software (SAS Institute, Cary, NC,). Sample size was determined by previous studies using US in a rat model of sciatic nerve crush injury [10,12]. Data are shown as mean ± SD. The differences among the groups were evaluated using the Student’s t-tests or one-way analysis of variance, and post hoc Tukey-Kramer tests were used. All continuous data were assessed for normal distribution using the Shapiro–Wilk normality test and for homoscedasticity using Bartlett’s test. P-values < 0.05 were considered statistically significant. In this study, “n” represents the number of independent observations from different rats per group.

## Results

### The intensity of ultrasound affected sciatic nerve regeneration

**Fig 2:**
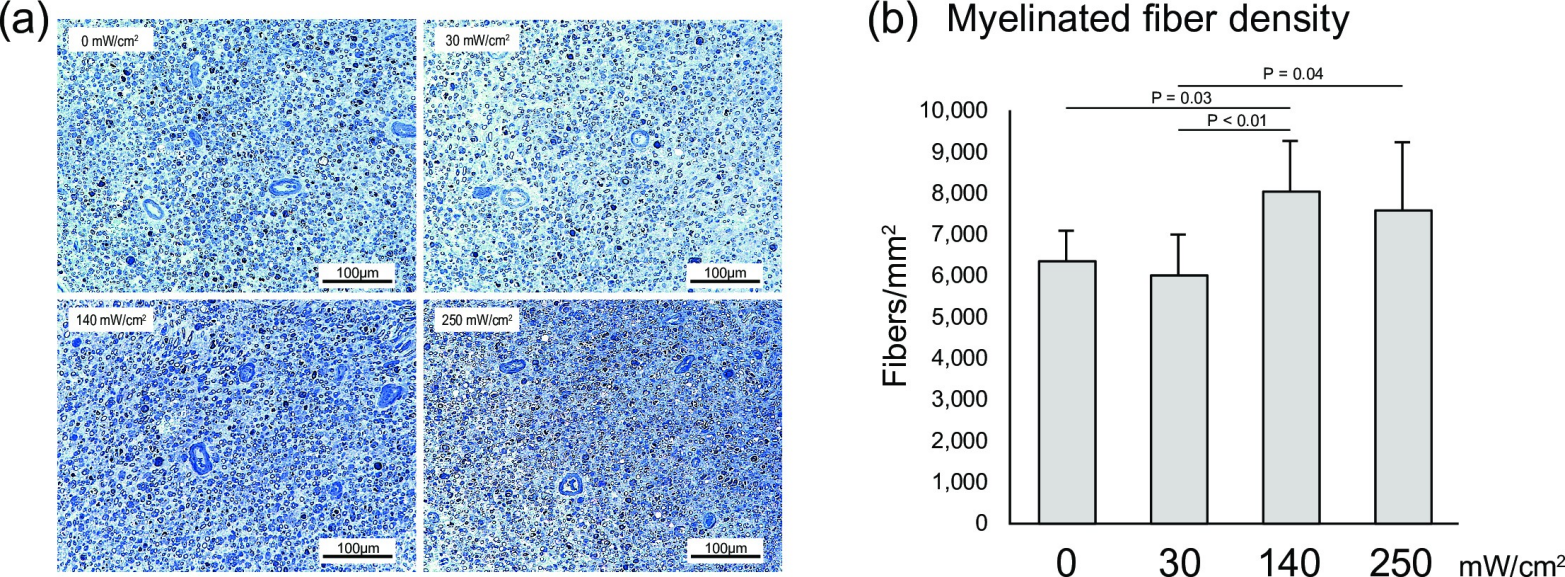
Myelinated fiber density. (a) Representative images of the sections stained with toluidine blue. (b) Mean myelinated fiber density. All data are presented as mean ± SD (n = 9).

**Fig 3:**
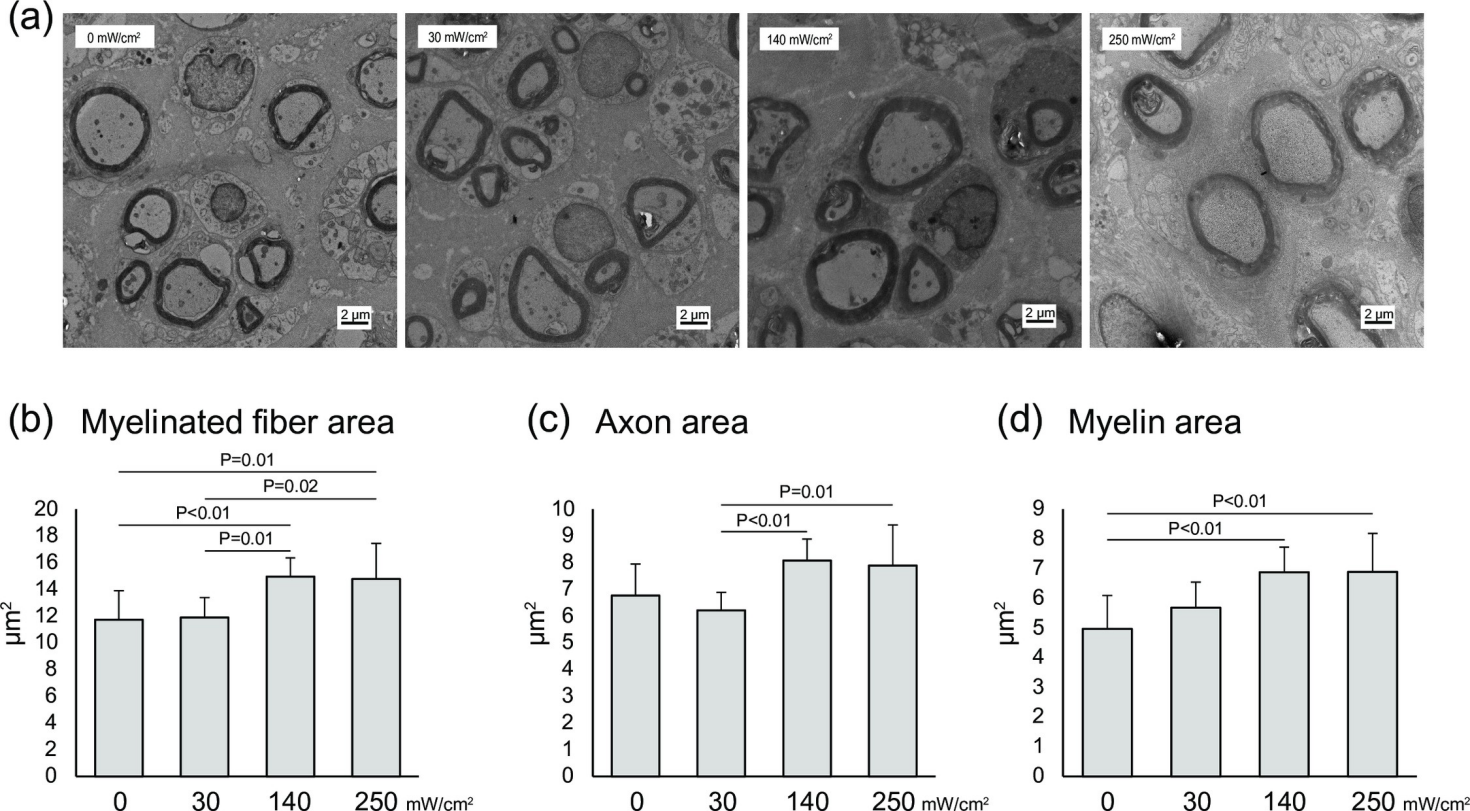
Transmission electron micrographs of the sciatic nerve. (a) Representative images of the transmission electron micrograph of the sciatic nerve. (b) Mean myelinated fiber area, (c) mean axon area, (d) mean myelin area. All data are presented as mean ± SD (n = 9).

Representative images of the nerve fibers stained with toluidine blue are shown in Fig 2A. Myelinated nerve fibers were visible in all groups since peripheral nerves have a spontaneous regeneration ability following axonotmesis. Quantitative analysis showed that the myelinated fiber density was significantly higher in the 140 mW/cm^2^ group than in the 0 mW/cm^2^ group (8037 fibers/mm^2^ ± 1226.2 vs. 6349 fibers/mm^2^ ± 742.9; P = 0.03) (Fig 2B). To evaluate nerve regeneration, ultrathin transverse sections of the nerve was observed by TEM. Representative images obtained by TEM are shown in Fig 3A. Quantitative analysis showed that the myelinated fiber area and myelin area were significantly greater in the 140 mW/cm^2^ and the 250 mW/cm^2^ groups than in the 0 mW/cm^2^ group (Myelinated fiber area, 140 mW/cm^2^: 15.0 μm^2^ ± 1.39, 250 mW/cm^2^: 14.8 μm^2^ ± 2.67, 0 mW/cm^2^: 11.7 μm^2^ ± 2.17; P < 0.01, P = 0.01, respectively. Myelin area, 140 mW/cm^2^: 6.9 μm^2^ ± 0.84, 250 mW/cm^2^: 6.9 μm^2^ ± 1.30, 0 mW/cm^2^: 5.0 μm^2^ ± 1.13; P < 0.01) (Fig 3B and 3D). However, there were no significant differences between the sham and US groups in terms of the axon area (Fig 3C).

### Sciatic functional index

**Fig 4:**
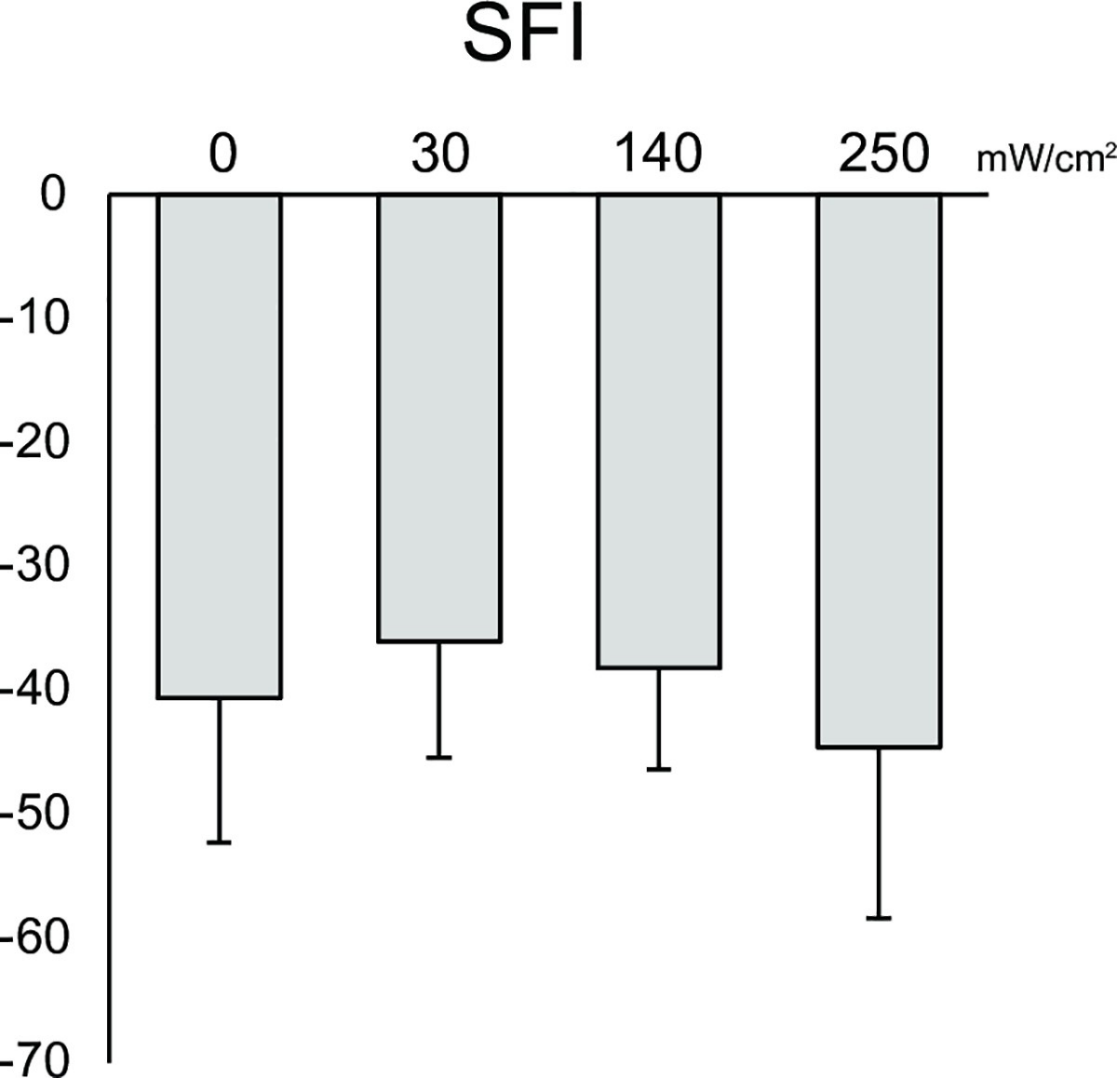
Sciatic functional index. All data are presented as means ± SD (n = 9). A value of 0 implies normal function and a value of -100 indicates total impairment.

To assess the effect of US for a motor function, SFI was evaluated. There were no significant differences among the groups (Fig 4).

### US stimuli inhibited mRNA expression of pro-inflammatory cytokines and axonal growth inhibitors

**Fig 5:**
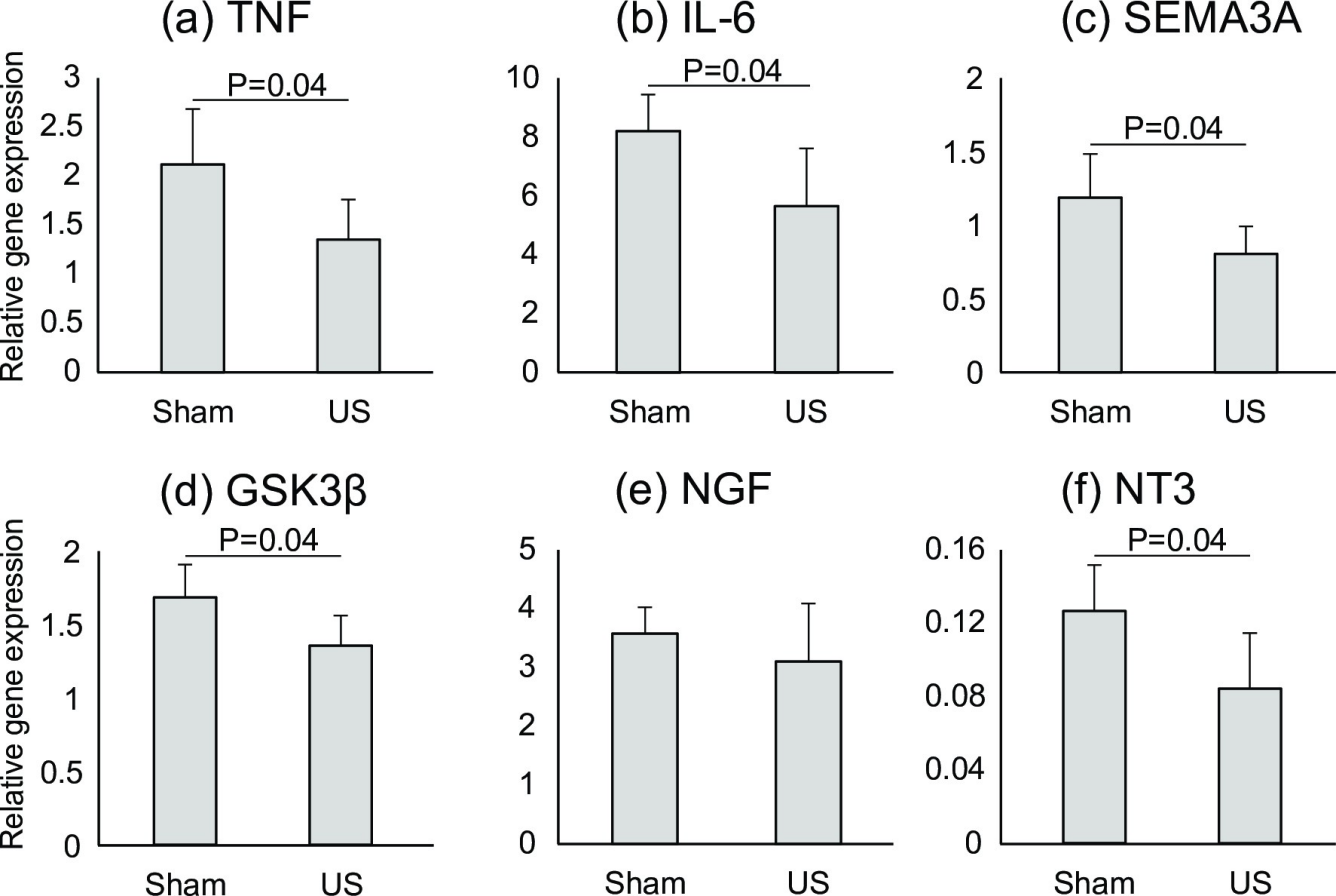
Gene expression analysis at 1 week after injury. Gene expression of (a) TNF, (b) IL-6, (c) SEMA3A, (d) GSK3β, (e) NGF, and (f) NT3 in the sham (0 mW/cm^2^) and US (140 mW/cm^2^) groups 1 week after injury. All data are presented as means ± SD (n = 5). The value of the calibration sample (intact nerve specimen) was set to 1.

Based on these intensity experiment results, we found that 140 mW/cm^2^ intensity was optimal in the present study condition. In order to reduce animal number used, we utilized only 0 mW/cm^2^ and 140 mW/cm^2^ groups for gene expression analysis to identify the mechanisms of nerve regeneration affected by US 1 week after the injury (Fig 5). Our results showed that US inhibited mRNA expression of the pro-inflammatory cytokines tumor necrosis factor (TNF) (Fig 5A) and interleukin 6 (IL-6) (Fig 5B) (P = 0.04) as well as those of the axonal growth inhibitors SEMA3A (Fig 5C) and glycogen synthase kinase 3 beta (GSK3β) (Fig 5D) (P = 0.04). In addition, we measured the expression of the neurotrophins NGF (Fig 5E) and neurotrophin 3 (NT3) (Fig 5F). The expression of NGF was up-regulated and that of NT3 was down-regulated in both sham and US groups compared to that in the intact condition (the value of the intact group was set as 1). In comparison with the sham and US groups, US significantly inhibited the expression of NT3 (P = 0.04) although there was no significant difference in the expression of NGF.

## Discussion

In this study, we investigated the effect of several US intensities on the regeneration of the sciatic nerve following a crush injury in rats and we explored the underlying mechanisms affected by US by analyzing mRNA expression. We found that the intensity of 140- and 250 mW/cm^2^ significantly accelerated re-myelination rather than the sham stimulation. Moreover, the density of myelinated fibers was significantly higher in the 140 mW/cm^2^ group than in the sham group, indicating that sprouting of the axons was promoted by US stimulation. In addition, US also induced down-regulation of mRNA expression of the inhibitors of axonal growth and myelination as well as pro-inflammatory cytokines. To the best of our knowledge, this finding is the first report regarding the effects on peripheral nerve regeneration.

Until now, few reports have discussed the effect of US intensity on the regeneration of peripheral nerves. To describe US intensity, SATA and SATP (spatial average and temporal peak) are commonly used. We strongly recommend that an author clearly states the notation of ultrasound intensity in their article. Otherwise, comparative study on intensity will be difficult [24]. Here, we use SATA unless otherwise specified to avoid confusion. Hong et al. [13] compared the US intensity of 100 mW/cm^2^ to that of 200 mW/cm^2^ using a rat sciatic nerve crush injury model. They found that the intensity of 100 mW/cm^2^ quickens the recovery of conduction velocity, while 200 mW/cm^2^ delays the recovery of conduction velocity compared to the control group. Mourad et al. [14] discussed four experimental conditions and found that an intensity of 250 mW/cm^2^ (spatial peak and temporal average) and a frequency of 2.25 MHz for 1 min enhanced the recovery of SFI the most. Comparing twenty conditions, Akhlaghi et al. [15] found the most effective US parameters for SFI recovery were the following: intensity, 100 mW/cm^2^; frequency, 1 MHz; pulse mode, duty cycle 20%; duration, 2 min. These previous studies reported incomplete outcomes such as nerve conduction velocity or SFI only. Using an autologous nerve transplantation model, Jiang et al. [11] applied three intensities, 250, 500, and 750 mW/cm^2^, and found that the lowest intensity was the most regenerative. However, the intensity of 250 mW/cm^2^ may still be too strong because most of the *in vivo* research showed satisfactory results when applying intensities of 15–200 mW/cm^2^ for the treatment of peripheral nerve diseases. Based on this, we hypothesized that an intensity lower than 250 mW/cm^2^ would have a more pronounced effect on nerve regeneration and set 250 mW/cm^2^ as the maximum intensity. In *in vitro* research, the effects of US on induced pluripotent stem cell-derived neural crest stem cells (iPSs-NCSCs) was investigated using 20–300 mW/cm^2^ intensity and found that 100 mW/cm^2^ facilitated viability, proliferation, and neural differentiation of iPSs-NCSCs without inducing any cytotoxicity [25]. These previous studies clearly indicate that an US with intensities of 100–250 mW/cm^2^ has a neuromodulator effect. Similarly, our study found that a 140 mW/cm^2^ intensity induced the most favorable regeneration, even though there is no positive effect at an intensity of 30 mW/cm^2^. This result is consistent with previous reports [13,15,26]. In addition, we showed that an intensity of 250 mW/cm^2^ induced nerve re-myelination to a similar degree as the 140 mW/cm^2^ intensity.

US has thermal and non-thermal effects. With regards to the regeneration of the peripheral nerve, it has been reported that the non-thermal effects are significant. Most US research uses a low-intensity pulse to maintain non-thermal effects on peripheral nerve regeneration. Micromechanical stimulation induces the non-thermal effects of US. This mechanical stress enhances enzyme activity through various mechanisms such as an increase in intra-cellular calcium concentration [27], resulting in an exacerbation of cellular metabolism. US-induced mechanical stress increases incorporation of trophic factors by enhancing the permeability of the cell membrane [28] and promotes synthesis of nitric oxide by endothelial cells [29]. These non-thermal effects of US at the cellular level may affect tissue regeneration including that of the peripheral nerve.

In the present study, US suppressed the mRNA expression of TNF, IL-6, SEMA3A, and GSK3β one week after injury. Sciatic nerve injury induces a rapid production and release of pro-inflammatory cytokines such as TNF and IL-6 [30]. Although these pro-inflammatory cytokines are essential in the early phase of Wallerian degeneration [31], they are attenuated until one week after the injury. After that, anti-inflammatory cytokines are predominantly released [30]. Pro-inflammatory cytokines act as initiators of Wallerian degeneration by activating resident Schwann cells and facilitating macrophage recruitment to the injury site [31]. Facilitation of the degenerative cascade after peripheral nerve injury results in early regeneration. Additionally, pro-inflammatory cytokines directly regulate nerve regeneration. For instance, TNFα inhibits neurite outgrowth [32,33]. Kato et al. [34] reported that immediate anti-TNFα therapy enhances axonal regeneration after a sciatic nerve crush injury in rats. Therefore, it is possible that US facilitates Wallerian degeneration by regulating pro-inflammatory cytokines.

SEMA3A contributes to the inhibition of axonal regeneration by binding with neuropilin-1 on growth cones. Omoto et al. [35] reported that the inhibitor SEMA3A promoted the regeneration of peripheral nerves in a mouse corneal transplantation model. GSK3β is a multifunctional serine/threonine kinase found in eukaryotes and thought to be a central regulator of the outgrowth of neurites [36,37]. The local inhibition of GSK3β enhances neurite elongation, whereas its overexpression impairs elongation [38,39]. Ren et al. [40] examined the effects of low-intensity pulsed US using rat cortical neurons and found that US enhanced elongation of neurites through inhibition of GSK3β, a finding which is consistent with our results. Based on these findings, US may accelerate axonal elongation through, in part, the suppression of pro-inflammatory cytokines and inhibitors of axonal elongation.

Peripheral nerve regeneration was also achieved by several neural trophic factors. In this study, NGF was up-regulated and NT3 was down-regulated by nerve injury compared to intact controls, consistent with previous studies [41,42]. However, US had no significant effect on the expression of NGF, but we noted a significant suppression of NT3. Chen et al. [10] found that regeneration of the sciatic nerve was facilitated by US with a 250 mW/cm^2^ intensity. They also reported that up-regulation of NGF expression is one of the mechanisms of action of US since the expression of NGF was increased by US 4 weeks after the injury. In the present study, we analyzed mRNA expression just one week after the injury since we focused on the anti-inflammatory effects of US which may be the reason we did not observe US-induced changes in NGF. NT3 is a member of the neurotrophin family and it has been reported to have an effect on neuronal survival and differentiation [43]. NT3 has other effects including the prevention of re-myelination [44]. For glial proliferation, elongation, and ensheathment, the expression of NT3 decreases whereas tropomyosin receptor kinase C (TrkC) receptors (which are responsible for NT3 inhibition of re-myelination) remains constant [45]. One of the possible mechanisms by which US accelerates re-myelination is the suppression of the mRNA expression of NT3. However, *in vitro* studies have shown that US at an intensity of 100 mW/cm^2^ up-regulated NT3 expression [46]. Further studies are needed to clarify the effects of US on the expression of NT3.

Conversely, no significant differences among the groups with regards to SFI were noted although there were significant differences with regards to histomorphometric parameters in this study. This discrepancy between the motor function and histology might be caused by inadequate methodology of motor function analysis. The values of the SFI begin to recover 2 weeks after the injury, rapidly improve after another one week and finally return to normal levels within 4 weeks [20]. Because we measured the SFI at only 3 weeks after the injury, it may have been too late to determine the effects of US treatment. More thorough evaluations are needed to clarify the effects of US on motor function [47].

There are several limitations to this study. Firstly, we could not completely exclude a thermal effect of US since we did not measure the temperature around the target tissue. However, Jiang et al. [11] reported that there was no temperature increase when even an intensity of 750 mW/cm^2^ was used. Therefore, we believe that there is no or negligible thermal effects since we utilized no more than one-third of the intensity compared to previous study. Secondly, we have not evaluated their sensory functions. The sciatic nerve contains motor and sensory fibers. In order to create functional and efficient movement, feedback from sensory nerves is necessary. However, few studies have examined the effects of US on sensory nerves. Further studies are warranted on the sensory function. Thirdly, we analyzed mRNA expression only one week after the injury. Therefore, the effects of US in the other phase could not be clarified in this study. Further, we did not analyze the mRNA expression in different intensity groups. Therefore, the underlying mechanisms in other intensity groups remain unclear. Finally, the intensity of US we determined cannot be applied in clinical studies because the optimum intensity may vary according to the species, model of injury, and thickness of the soft tissue. US is attenuated by superficial tissues. We need to confirm the effects of US via pre-clinical studies using a larger number of animals with a tissue architecture similar to that of humans.

## Conclusions

We attempted to identify the optimum intensity of US required to promote peripheral nerve regeneration after crush injury in rats and explore the underlying mechanisms associated with US therapy. We concluded that 140 mW/cm^2^ was the optimum intensity in this study condition and that the suppression of the pro-inflammatory and nerve growth inhibitor gene expressions would be, in part, attributed to this effect.

## Supporting information

S1 Fig Ranking of the candidate reference genes. A stable endogenous reference gene was determined by exploring 32 candidate genes using a TaqMan® Array Rat Endogenous Control plate. (a) comprehensive gene stability was highest for proteasome 26S subunit, ATPase, 4 (Psmc4) in both RefFinder and NormFinder. (b) The standard deviation (SD) of each candidate reference gene among different subgroups (intact, sham, and 140 mW/cm^2^ groups) is shown. The intra-subgroup SD is indicated by the vertical bars. Psmc4 showed the smallest variation among the genes.
(TIF)

S1 Dataset Dataset for supporting information. (XLSX)

S1 File Completed “The ARRIVE Guidelines Checklist” for reporting animal data. (PDF)
