# Supplementary figures and images for: Ultrasound therapy with optimal intensity facilitates peripheral nerve regeneration in rats through suppression of pro-inflammatory and nerve growth inhibitor gene expression
Source: PLoS One. 2020 Jun 17;15(6):e0234691. doi: 10.1371/journal.pone.0234691 (PMC7299378; doi:10.1371/journal.pone.0234691)

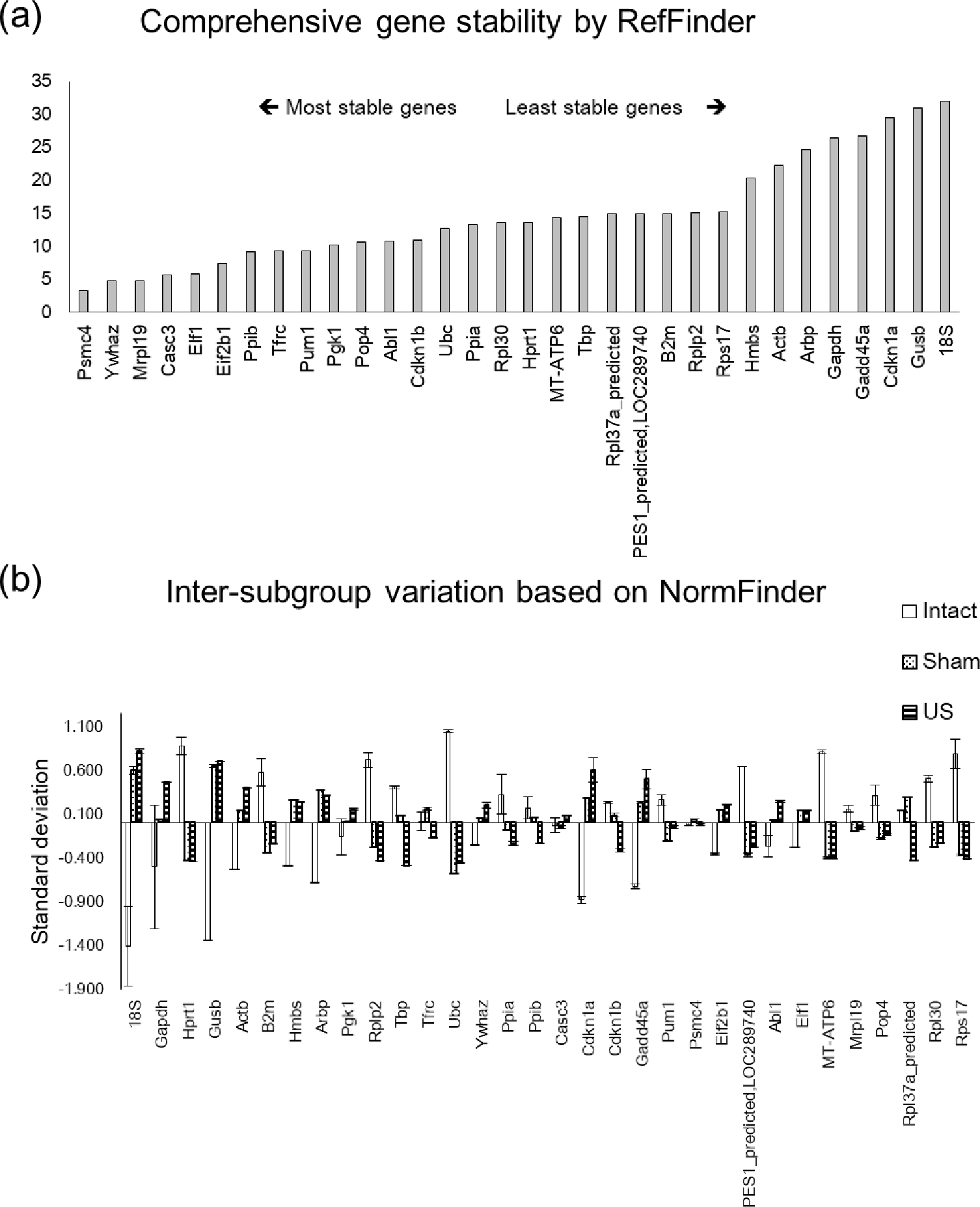

Supplement: S1 Fig — A stable endogenous reference gene was determined by exploring 32 candidate genes using a TaqMan® Array Rat Endogenous Control plate. (a) comprehensive gene stability was highest for proteasome 26S subunit, ATPase, 4 (Psmc4) in both RefFinder and NormFinder. (b) The standard deviation (SD) of each candidate reference gene among different subgroups (intact, sham, and 140 mW/cm2 groups) is shown. The intra-subgroup SD is indicated by the vertical bars. Psmc4 showed the smallest variation among the genes. (TIF) [file pone.0234691.s001.tif]
